# Monitoring T Cells Responses Mounted by Therapeutic Cancer Vaccines

**DOI:** 10.3389/fmolb.2021.623475

**Published:** 2021-04-15

**Authors:** Kue Peng Lim, Nur Syafinaz Zainal

**Affiliations:** Cancer Immunology and Immunotherapy Research Unit, Cancer Research Malaysia, Subang Jaya, Malaysia

**Keywords:** cancer vaccine, immunotherapy, T cell response, tetramer, immune monitoring, immune activation

## Abstract

With the regulatory approval of Provenge and Talimogene laherparepvec (T-VEC) for the treatment of metastatic prostate cancer and advanced melanoma respectively, and other promising clinical trials outcomes, cancer vaccine is gaining prominence as a cancer therapeutic agent. Cancer vaccine works to induce T cell priming, expansion, and infiltration resulting in antigen-specific cytotoxicity. Such an approach that can drive cytotoxicity within the tumor could complement the success of checkpoint inhibitors as tumors shown to have high immune cell infiltration are those that would respond well to these antibodies. With the advancements in cancer vaccine, methods to monitor and understand how cancer vaccines modify the immune milieu is under rapid development. This includes using ELISpot and intracellular staining to detect cytokine secretion by activated T cells; tetramer and CyTOF to quantitate the level of antigen specific T cells; proliferation and cell killing assay to detect the expansion of T cell and specific killing activity. More recently, T cell profiling has provided unprecedented detail on immune cell subsets and providing clues to the mechanism involved in immune activation. Here, we reviewed cancer vaccines currently in clinical trials and highlight available techniques in monitoring the clinical response in patients.

## Introduction

Vaccination against infectious diseases such as smallpox, polio, mumps and measles have markedly reduced mortality rates from these diseases and have been adopted as a standard practice across the world. The idea of cancer vaccination was first discovered in late 1800, when Dr. William Coley demonstrated better patient outcomes after the administration of Coley toxin. However, due to the absence of good manufacturing protocol, this approach yielded varied outcomes as different institutions used different formulations. Notably, the mechanism of action of Coley toxin was not fully understood, as the knowledge underlying immune activation was limited at that time. Radiation therapy was introduced at the same time and this demonstrated clear and consistent results in all patients, hence the development of Coley toxin was neglected. Only until recently, to further improve the success of immunotherapy in the form of checkpoint inhibitors, the interest in using vaccination to treat cancer is reinvigorated. This is mainly due to the ability of vaccines to stimulate the proliferation of antigen specific T cells, the main effector cells in fighting and killing cancer cells. Generally, two approaches have been developed, the first, is to use attenuated cancer cells or unique antigens (in the form of neoantigen, mRNA, DNA, peptide, or dendritic cell) expressed by cancer cells to stimulate immune responses to recognize cancer cells; the second approach is to generate immune response *in situ* using oncolytic viruses (either virus alone or virus-expressing cancer antigens) that selectively replicate in tumor tissue, which will then enhance antigen presentation and different immune responses (Conry et al., 2018).

Cancer vaccine is predicted to be one of the fastest growing areas in immunotherapy development worldwide. To investigate the up-to-date development in the cancer vaccine field, we have conducted a thorough search using the ClinicalTrials.gov database. Using “cancer” as disease and “cancer vaccine” as other terms, yielding 1326 interventional studies after excluding studies that are suspended, terminated, withdrawn, with unknown status or involved children. From these 1326 studies, we further sub-classified cancer vaccine into seven types according to the format of the cancer vaccine at the time of administration, (1) neoantigen, (2) mRNA, (3) DNA, (4) peptide, (5) dendritic cell, (6) cellular vaccines, and (7) oncolytic virus (Table 1). Notably, cancer vaccines in the form of peptide and dendritic cell have the highest number of on-going or completed trials. We also noted some overlapped due to the similarity of the format of the vaccine, for example, neoantigen vaccine can also be classified as peptide vaccine as the mutated epitope was actually a string of amino acids. We focus on the newly published clinical trials on cancer vaccines and their respective immune monitoring approaches in subsequent sections.

**TABLE 1:** Types of cancer vaccine that are having on-going or completed clinical trials.

**Vaccine type**	**Search term**	**Hits**	**Cancer involved**
Neoantigen vaccine	Cancer vaccine, neoantigen	47	Leukemia, breast CA, liver, lung, ovarian, renal, head and neck, colon, myeloma, pancreas, prostate, skin, urogenital
mRNA vaccine	Cancer vaccine, mRNA	41	Brain, breast, liver, lung, ovarian, renal, colon, esophageal, head and neck, leukemia, kidney, lymphoma, melanoma, pancreas, prostate, rectal, urogenital
DNA vaccine	Cancer vaccine, DNA	117	Brain, breast, Merkel cell, lung, ovarian, renal, cervical, colon, esophageal, gall bladder, head and neck, kidney, nasopharyngeal, pancreas, stomach, skin, thyroid, urogenital
Peptide vaccine	Cancer vaccine, peptides	347	Anal, brain, Hodgkin lymphoma, breast, leukemia, kidney, liver, lung, ovarian, renal, colon, esophageal, gallbladder, laryngeal, myeloma, nasopharyngeal, osteosarcoma, ovarian, pancreas, prostate, rectal, head and neck, skin, stomach, thyroid, cervical, urogenital
Dendritic cell vaccine	Cancer vaccine, dendritic cells	271	Lung, breast, myeloma, ovarian, lymphoma., leukemia, bill duct, brain, liver, renal, colon, esophageal, gall bladder, kidney, nasopharyngeal, pancreas, prostate, testicular, urogenital, thyroid
Cellular vaccine	Cancer vaccine, allogenic OR Allostim OR GVAX	113	Leukemia, breast, lung, ovarian, renal, myeloma, colon, head and neck, Hodgkin lymphoma, liver, lymphoma, melanoma, pancreas, prostate, rectal, skin, urogenital
Oncolytic virus	Cancer vaccine, oncolytic virus	14	Ovarian, breast, lung, brain, mesothelioma, myeloma, melanoma
MISC	–	375	–
Total		1326	

## The Landscape of Cancer Vaccines

### DNA/RNA/Peptide-Based Vaccine

DNA/RNA/peptide-based vaccines are different vaccine formats of how antigens are presented or delivered into the body to elicit antigen-specific immune responses. All of which have their advantages and disadvantages; DNA is stable and can be easily manipulated (codon-optimization, adding on sequences that could code for immune adjuvants, etc.) and produced. Whilst RNA is similar to DNA, it is less stable and requires a cold-chain for transportation. However, both DNA and RNA have limited delivery efficacy (Jou et al., 2021). Peptide vaccines are peptides of 9–25 amino acid in length designed based on the prediction of specific regions of an antigen that can bind to MHC-I and II molecules. As compared to DNA and RNA vaccine, peptide vaccine is safer as it does not contain any extraneous regions like promoter or antibiotic resistance regions in the expression vector but the usage is often limited to patients with specific MHC subtypes. Table 2 presents an overview of the DNA/RNA/peptide vaccines that have resulted in a publication from 2018 to 2020.

**TABLE 2:** DNA/RNA/peptide-related cancer vaccine.

**Name**	**Composition**	**Indication**	**Trial design (single-arm unless otherwise is stated) and outcome**	**Immune monitoring method/outcome**	**References**
pTVG-HP (MVI-816)	DNA vaccine encoding prostatic acid phosphatase	Phase 2: Progressive, non-metastatic, castration-sensitive prostate cancer	2-arm: (i) pTVG-HP vaccine with GM-CSF (ii) GM-CSF alone 2-year metastasis-free survival (MFS) rate (i) 41.8% (ii) 42.3% *p* = (0.97)	**Fluorospot:** No significant differences in PAP-specific IFN-γ release were observed over time in vaccine-treated patients. PAP-specific multifunctional Th1-biased T cells (secreting IFN-γ, TNF-α, and granzyme B) were significantly increased at month 3 in patients who received pTVG-HP up to month 6.	NCT01341652 (McNeel et al., 2019)
pTVG-HP DNA vaccine	DNA vaccine encoding prostatic acid phosphatase	Phase 2: Patients with castrate-resistant, metastatic prostate cancer	2-arm: (i) Sipuleucel-T alone (ii) Sipuleucel-T + pTVG-HP DNA vaccine Median TTP was less than 6 months and not statistically different between study arms.	**IFN-y/granzyme B ELISpot:** Th1-biased PAP-specific T-cell responses were detected in 11/18 individuals, and were not statistically different between study arms.	NCT01706458 (Wargowski et al., 2018)
GX-188E (HPV DNA vaccine)	GX-188E is a HPV E6/E7 DNA therapeutic vaccine, consisting of a tissue plasminogen activator signal sequence, and FMS-like tyrosine kinase 3 ligand, and shuffled E6 and E7 genes of HPV type 16/18	Phase 2: cervical intraepithelial neoplasia	2-arm (i) 1 mg GX-188E (ii) 4 mg GX-188E Histopathologic regression to ≤CIN1 among 72 patients, 52% (visit 7) and 67% (visit 8) showed regression.	**IFNγ ELISpot:** Patients with regression had significantly higher immune response against GX-188E vs. patients without regression	NCT02139267 (Choi et al., 2020)
MEDI0457 DNA vaccine	DNA immunotherapy targeting HPV16/18 E6/E7 with IL12 encoding plasmids	Phase 1/2a: patients with HPV-associated locally advanced HNSCC	Immunotherapy was safe and well tolerated in both settings (cohort i and ii). No serious AEs were reported.	**IFN-γ ELISpot:** 18/21 evaluable patients showed elevated antigen-specific T-cell activity and persistent cellular responses surpassing 100 spot-forming units (SFUs)/106 peripheral blood mononuclear cells (PBMCs) were noted out to 1 year **IHC:** MEDI0457 modulates immune infiltration into tumor tissues **Flow-cytometric analyses:** MEDI0457 induces antigen-specific cytotoxic T cells, induce HPV16-specific PD-1 + CD8+ T cells that were not found before MEDI0547 (0% vs. 1.8%) **ELISA:** MEDI0457 induces the generation of HPV16- and HPV18-specific antibodies. All patients showed induction of humoral responses against at least 1 HPV-specific antigen, with persistence of humoral responses for up to 23 months.	NCT02163057 (Aggarwal et al., 2019)
National Cancer Institute (NCI)-4650 RNA vaccine	Up to 15 predicted neoantigens were selected based on WES and RNASeq and their binding affinity to the patients’ HLA molecules Sequences composed of 25 aa with the mutation flanked by 12 normal aa on each side were electronically submitted to Moderna Therapeutics for the manufacturing of a TMG based vaccine	Phase 1/2: metastatic gastrointestinal (GI) cancer	The vaccine is safe and the maximum tolerated dose (MTD) was not reached. No objective clinical responses in the four patients treated in this trial	**IFN-γ ELISpot:** Detected CD8 and CD4+ neoantigen-specific T cells elicited by the vaccine in 3/4 patients. **Flow cytometry:** A human soluble cluster of differentiation 137 (CD137) (4-1BB) was upregulated against certain epitopes.	NCT03480152 (Cafri et al., 2020)
FixVac (BNT111) RNA vaccine	Melanoma FixVac is composed of RNA-LPX encoding four TAAs (NY-ESO-1, MAGEA3, tyrosinase, and TPTE)	Phase 1: stage III B-C or IV melanoma	2-arm: (i) FixVac alone (ii) FixVac+ anti-PD1 The clinical adverse-event profile was dominated by mild to moderate flu-like symptoms, such as pyrexia and chills	**IFN-γ ELISpot:** *Ex vivo*: more than 75% showed immune responses against at least one TAA. **Intracellular cytokine staining:** Samples from all 20 of these patients showed a T-cell response against at least one TAA.	NCT02410733 (Sahin et al., 2020)
Personalized peptide vaccination (PPV)	Four of 12 warehouse peptides selected based on pre-existing peptide-specific immunoglobulin G levels	Phase 3: Recurrent glioblastoma	2-arm (i) PPV (ii) Placebo The trial met neither the OS nor secondary endpoints.	**IFN-γ ELISpot:** CTL activities specific to at least one of the four vaccinated peptides were boosted at least once throughout the vaccination in 68% of PPV post-vaccinated patients. **Luminex assay:** IgG boosting was observed in the post-vaccination plasma from 54% of PPV patients.	Narita et al. (2019)
VEGFR1 and 2 vaccines	VEGFR1-A24–1084 and VEGFR2-A24–169 peptide (2 mg of each) emulsified together with 1 ml of incomplete Freund’s adjuvant + temozolomide (TMZ) -based chemoradiotherapy	Phase 1/2: Primary glioblastoma	No grade 3/4 AE was found. 50% achieved a complete response.	**IFN-γ ELISpot:** CTLs specific for both VEGFR1 and 2 were induced after vaccination in 2/4 patients. **Multiplex IF:** In the post-vaccination tumor, expression of cleaved caspase 3 was co-localized in endothelial cells with CD34- and Foxp3-positive cells. No changes in the number of CD163, CD8, and CD4-positive cells after vaccination.	Tamura et al. (2020)
5 peptides vaccine	5 HLA-A*24:02 restricted epitope-peptides: RNF, TOMM, KOC1 (IMP-3), VEGFR1 and VEGFR2 + oxaliplatin-containing chemotherapy	Phase 2: Unresectable metastatic colorectal cancer	VEGFR2 IgG responses may be an important immunological biomarker in the early course of treatment for CRC patients treated with therapeutic epitope peptides	**Multiplex bead suspension Luminex:** Plasma levels of TOMM34 IgG, RNF43 IgG and VEGFR2 IgG were significantly increased after vaccination. Stronger VEGFR2 IgG responses correlated significantly with OS in HLA-matched patients. **IFN-γ ELISpot:** CTL responses to VEGFR1 and VEGFR2 were significantly increased in the HLA-matched group. No correlation of increased CTL response with OS.	Kanekiyo et al. (2018)
RNF43 and TOMM34 peptide vaccine	RNF43 and TOMM34 peptide vaccine + uracil-tegafur (UFT/LV) chemotherapy	Phase 2: Metastatic colorectal cancer	In the HLA-matched group, 3-year relapse-free survival (RFS) was significantly better in the positive CTL subgroup than in the negative-response subgroup.	**IFN-γ ELISpot:** In the HLA-matched group, 14/28 patients had positive antigen-specific CTL responses after two cycles of treatment and 9 had negative responses; in the HLA-unmatched group, 10/16 CTL responses were positive and 2 negatives.	Kawamura et al. (2018)
Mixed 20-peptide cancer vaccine (KRM-20)	20 peptides originating from twelve different TAAs, including PSA, PAP, PSMA, EGF-R, PTHrP, SART3, CypB, WHSC2, UBE2V, HNRPL, p56lck, and MRP3	Phase 2: Castration-resistant prostate cancer	2-arm: (i) KRM-20 combined with docetaxel and dexamethasone (ii) Placebo with docetaxel and dexamethasone Similar PSA decline in the two arms.	**Luminex system:** HLA-matched peptide-specific IgG responses in the KRM-20 arm significantly increased after treatment. **IFN-γ ELISpot:** HLA-matched peptide-specific CTL responses in the KRM-20 arm significantly increased after treatment.	Noguchi et al. (2020)
ISA 101	ISA 101 (long-peptide HPV-16 vaccine) + Nivolumab	Phase 2: incurable HPV-16-positive cancer	Of the 24 patients, ORR was 33% (8 patients; 90% CI, 19%–50%). The median duration of response was 10.3 months (95% CI, 10.3 months to inestimable). 5/8 patients remain in response.	**IFN-γ ELISpot:** An increase in the number of HPV-specific T cells was observed in both responders and non-responders after vaccination.	NCT02426892 (Massarelli et al., 2019)
Multivalent WT1 peptide vaccine (galinpepimut-S)	WT1 peptide vaccine	Phase 2: Acute myeloid leukemia in first complete remission (CR1)	Median disease-free survival from CR1 was 16.9 months. WT1 vaccine was well tolerated.	**T-cell proliferation:** Four of nine patients tested had detectable CD4 immune responses. **IFN-γ ELISPOT/tetramer assay:** Six of seven patients had a positive CD8 immune response against WT1-A peptide	NCT01266083 (Maslak et al., 2018)
Mixed 19−peptide vaccine	19 peptides derived from 11 different TAAs, including SART3, CypB, WHSC2, UBE2V, HNRPL, Lck and MRP3, PSA, PAP, EGFR, and PTHrP	Phase 2: advanced metastatic triple-negative breast cancer (mTNBC) refractory to systemic chemotherapy	No severe AE was reported. The median OS was 11.5 or 24.4 months in all 14 patients or the 10 patients who completed the vaccination.	**Luminex system:** Postvaccination peptide-specific IgG levels showing at least a 2−fold increase compared to pre-vaccination. **IFN-γ ELISpot:** Vaccination induced positive peptide-specific CTL responses in 5/10 patients.	Toh et al. (2020)
VEGFRs peptide vaccine	Peptides for VEGF1 and VEGF2	Phase 1/2: progressive neurofibromatosis type 2	No severe AE was reported. Hearing improves in 2/5 assessable patients (40%). Tumor volume reductions of ≥20% are observed in two patients.	**IFN-γ ELISpot:** CTLs specific for both VEGFR1 and VEGFR2 were induced after the vaccinations in six patients. Only CTLs specific for VEGFR1 were induced in one patient. Strong CTL responses against VEGFR2 were still detected 7 months post vaccine in three patients. **IHC** Tumor vessels exhibited negative or slight VEGFRs expression after vaccination. Levels of Foxp3-positive cells decreased after vaccination. No significant changes in total numbers of CD8-positive cells after vaccination, but observed increase in numbers of CD8-positive cells in the perivascular area. **Immunofluorescence** Many CD8-positive cells were observed around vessels with weak VEGFRs expression after vaccination	Tamura et al. (2019)
GPC3 peptide vaccine	GPC3 peptide vaccine	Phase 2: Glypican-3- positive hepatocellular carcinoma	Vaccination may reduce the 1-y recurrence rate and prolong overall survival in patients who are positive for GPC3 IHC staining.	**IFN-γ ELISpot:** CTL numbers ranged from 1 to 648 with a median of 27 after vaccination as compared to almost none before vaccination. **IHC:** Patients who were positive for GPC3 IHC staining were more likely to have induced CTLs	Taniguchi et al. (2020)
NY-ESO-1 protein	Full-length NY-ESO-1 protein + poly-ICLC + montanide^®^ ISA-51 VG	Phase 1/2: high-risk resected melanoma	For Phase 2 (2-arm): Arm A: NY-ESO-1 protein with poly-ICLC alone Arm B: NY-ESO-1 protein, poly-ICLC and montanide^®^ ISA-51 VG. Vaccine regimens were generally well-tolerated, with no treatment-related grade 3/4 adverse events.	**ELISA/epitope mapping:** All patients developed antibody responses to NY-ESO-1 protein. Antibody titers were not significantly different between patients in arms A and B), however, arm B developed more NY-ESO-1-specific antibodies compared to patients within arm A after three or four vaccines. T-cell avidity toward NY-ESO-1 peptides was higher in patients vaccinated with montanide. I**ntracellular cytokine staining (ICS):** After vaccination, NY-ESO-1-specific CD8+ T-cell responses were detected in 1 of 12 (8%) patients in arm A and 3 of 9 (33%) patients in arm B. Increase in IFN-γ production seen mainly in patients treated with montanide (Arm B).	NCT01079741 (Pavlick et al., 2020)
Adjuvant multi-peptide vaccine (UroRCC)	UroRCC is an adjuvant multipeptide vaccine	Phase 1/2: Metastatic renal cell carcinoma patients	Adverse events of UroRCC were mainly grade I and II. Median OS was not reached in the UroRCC group (mean: 112.6 months, 95% confidence interval [CI]: 92.1–133.1) and 58.0 months (95% CI: 32.7–83.2) in the control cohort.	**IFN-γ ELISpot:** The frequency of immune response was higher for MHC class II peptides (17/19, 89.5%) than for MHC class I peptides (8/19, 42.1%).	NCT02429440 (Rausch et al., 2019)
Survivin 2B peptide (SVN-2B)	In Step 1, the groups received treatments of: (i) survivin 2B peptide (SVN-2B) plus interferon-β (IFNβ); (ii) SVN-2B only; or (iii) placebo until the patients show progression. In Step 2, all patients who consented to participate received SVN-2B plus IFNβ.	Phase 2: Unresectable and refractory pancreatic carcinoma	No significant improvement in PFS was observed. Among patients who participated in Step 2, those who had received SVN-2B plus IFNβ in Step 1 showed better overall survival compared with those who had received placebo in Step 1.	**Tetramer assay:** Survivin 2B-specific CTLs were found to be increased in the SVN-2B plus IFNβ group. **IFN-γ ELISpot:** Patients vaccinated with SVN-2B plus IFNβ did not have improved PFS, but showed a significant immunological reaction after vaccination.	Shima et al. (2019)

The use of DNA vaccine encoding HPV16/18 in cancer prevention has remarkably caused more than 50% of patients with cervical intraepithelial neoplasia to experience lesion regression (Choi et al., 2020). Subsequently, this vaccine is also being evaluated as a therapeutic vaccine in HPV positive head and neck cancer and was shown to elicit strong T cells and B cells responses in majority of patients (Aggarwal et al., 2019). DNA Vaccine Encoding Prostatic Acid Phosphatase (pTVG-HP) has been tested in both castration-sensitive and castration-resistant prostate cancer. However, it did not demonstrate immunological and clinical benefits (Scurr et al., 2017; Wargowski et al., 2018). Recently, promising clinical response was observed in melanoma patients who failed anti-PD1 treatment by using the of RNA-lipoplex vaccine (targeting four tumor associated antigens (TAA) namely NY-ESO-1, MAGEA3, tyrosinase and TPTE; FixVac) (Sahin et al., 2020). This encouraging result has provided hope for patients who failed checkpoint immunotherapy. In addition to TAA, neoepitopes resulting from cancer-specific mutation are also a preferred target for cancer vaccine as the mutation is not present during the selection in the thymus and thus exempt from central tolerance (Wickström et al., 2019). The use of mRNA vaccine encoding up to 15 neoepitopes was evaluated in patients with metastatic gastrointestinal cancer where positive mutation-specific T cell responses were detected in patients after vaccination (Cafri et al., 2020). However, the use of neoantigen-specific vaccine that requires routine next-generation sequencing and the need for timely vaccine design and manufacture pipeline has hindered this from wide-scale implementation.

The use of MAGE-A3 peptide vaccine as cancer therapeutic agent has resulted in a big disappointment as no significant clinical benefit was observed in two Phase III clinical trials (MAGRIT and DERMA) for patients with non-small cell lung cancer and melanoma (Vansteenkiste et al., 2016; Dreno et al., 2018). To circumvent the lack of response to peptide vaccine, new studies are designed to target more than one antigen to avoid the risk for tumor escape due to antigen loss and to combine these vaccines with other approved therapies, for example, chemotherapy and checkpoint blockade inhibitors (Kanekiyo et al., 2018; Kawamura et al., 2018; Noguchi et al., 2020; Tamura et al., 2020). The use of RNF43/TOMM3 peptide vaccine in combination with chemotherapy in metastatic colorectal cancer has demonstrated significantly better 3-year relapse-free survival in patients who have higher T cell responses compared to the negative response subgroup (Kawamura et al., 2018). The use of HPV 16 synthetic long peptide in combination with nivolumab resulted in an overall response rate of 33% and median overall survival of 17.5 months in patients with oropharyngeal carcinoma, which is better than PD-1 inhibition alone in a similar patient cohort (Massarelli et al., 2019).

### Cellular-Based Vaccine

Cellular vaccine refers to cell-based vaccines that use autologous tumor cells or antigen-presenting dendritic cells to stimulate the immune system. Sipuleucel-T, an autologous dendritic cell targeting prostatic acid phosphatase (PAP) has improved survival for men with castration-resistant prostate cancer, is the first cancer vaccine approved by FDA in 2010 (Kantoff et al., 2010). To further improve the clinical response of Sipuleucel-T, DNA vaccine (pTVG-HP) targeting PAP was administered to patients who received Sipuleucel-T. Despite demonstrating higher antibody titer in patients who received pTVG-HP booster immunization as compared to Sipuleucel-T alone, the median time to progression was similar for both arms (Wargowski et al., 2018). Wilm’s tumor (WT1) protein is overexpressed in hematologic tumor and various solid tumors and it is recognized as one of the most promising targets for cancer immunotherapy (Cheever et al., 2009). Dendritic cell targeting WT1 has been evaluated in a number of early phase clinical trials where 7/10 bladder cancer patients, 7/10 breast/ovarian/gastric cancer patients, and 5/11 head and neck cancer patients achieved stable disease. Notably, patients who demonstrated clinical benefit developed higher WT1 specific cytotoxic T cell responses (Ogasawara et al., 2019; Zhang et al., 2019; Nagai et al., 2020). Recently, the use of genetically modified dendritic cells has been reported. Adenovirus was employed to deliver tumor-associated antigens (survivin, MUC1), secretory bacterial flagellin and an RNA interference moiety to suppress SOCS1 in dendritic cells (gmDC). This novel engineered gmDC was shown to be safe and resulted in 10/12 relapsed acute myeloid leukemia patients to experience complete remission (Wang et al., 2018).

The use of tumor cell lysate as vaccine is also a fast-growing field. The use of GVAX, a GMCSF secreting allogeneic pancreatic tumor cells has resulted in clinical response in metastatic pancreatic adenocarcinoma and is being tested in combination with CRS-207, a recombinant live-attenuated double-deleted Listeria monocytogenes (Lutz et al., 2011; Le et al., 2015; Nair et al., 2020). The use of dendritic cell presenting tumor lysate is also being evaluated in melanoma and colon cancer and data showed that this approach is well tolerated (Geskin et al., 2018; Rodriguez et al., 2018). Another study in renal cell carcinoma was using dendritic cell presenting tumor lysate with addition of systemic CpG and IFN-α. Encouragingly 6/15 patients achieved clinical response (1 complete response, 2 partial response, and 3 stable disease) (Koster et al., 2019). Table 3 presents an overview of the cellular vaccines that have resulted in a publication from 2018 to 2020.

**TABLE 3:** Cellular cancer vaccine.

**Name**	**Composition**	**Indication**	**Trial design (single-arm unless otherwise is stated) and outcome**	**Immune monitoring method/outcome**	**References**
Ad.p53-DC	Dendritic cells transfected with wild type p53	Phase 2: Small cell lung cancer completed first-line chemotherapy with stable disease, partial response, or complete response	3-arm (i) Observation + second line chemotherapy (ii) Ad.p53-DC + second line chemotherapy (iii) Ad.p53-DC + all *trans*-retinoic acid + second line chemotherapy ORR for treatment arms (i) 15.4% (ii) 16.7% (iii) 23.8%	**IFN-γ ELISpot against p53** (i) Not available (ii) 20.0% (iii) 43.3%	NCT00617409 (Chiappori et al., 2019)
Folate receptor loaded DC	Multi-epitope folate receptor alpha-loaded dendritic cell	Phase 1 (single arm): Stage IIIC-IV ovarian cancer patients in the first remission after conventional therapy.	No grade III and higher adverse event	**IFN-γ ELISpot to FRα:** = 89% (16/18) **ELISA:** (i) Antibody responses targeting native FRα protein: 50% (ii) Antibody responses to individual vaccine epitopes: 39–94%.	NCT02111941 (Block et al., 2020)
Tumor lysate-DC	Monocyte-derived dendritic cells preloaded with autologous tumor lysate	Phase 1: Treatment-refractory histologically confirmed solid tumors	Safe and well tolerated	**RT-PCR:** Increased IFN-b and IFN-a mRNA in circulating PBMC. **ELISA:** Increased serum IL-12 and IL-1b concentrations, especially in patients presenting stable disease. **IFN-γ- ELISpot:** IFNγ-ELISPOT reactivity to tumor lysates was observed in two patients experiencing durable stable disease.	Rodríguez-Ruiz et al. (2018)
Monocyte-derived dendritic cell (MODC) vaccines	Monocyte-derived dendritic cell (MODC) vaccines	Phase 2: Metastatic melanoma	3-arm: Dendritic cells with (i) Co-cultured with tumor cells (ii) Pulsed with tumor cells (iii) Fused with tumor lysate Safe and well-tolerated by all patients	**Delayed-type hypersensitivity:** 5/15 patients achieved delayed-type hypersensitivity responses. **IFN-γ ELISpot:** 6/15 patients had positive ELISpot results.	Geskin et al. (2018)
DC - tumor lysate	DC loaded with autologous tumor lysate	Phase 2: metastasis of colon adenocarcinoma	2-arm: (i) DC vaccine (ii) Observation Fewer and later relapses in the vaccine arm. The median disease-free survival was 25.26 months in the vaccination arm as compared to 9.53 months in the observation arm.	**IFN-γ ELISpot:** A clear increase in reactivity to tumor lysate-loaded DC was only seen in one of the non-relapsed cases. Marginal increases were observed in other cases	NCT01348256 (Rodriguez et al., 2018)
ATV	An irradiated autologous tumor cell vaccine (ATV) co-injected with a class-B CpG oligodeoxynucleotide (CpG-B) and GM-CSF, followed by systemic CpG-B and IFN-α administration	Phase 2: Metastatic renal cell carcinoma	Objective clinical responses occurred in three patients, including one long-term complete response. Disease stabilization occurred in another three patients.	**Delayed type hypersensitivity (DTH):** DTH in 13 out of 15 patients **IFN-γ ELISpot:** Autologous tumour cells recognizing circulating T cells were revealed to be more frequent in patients who clinically benefited from the therapy	Koster et al. (2019)
WT1-DC	WT1-peptide pulsed dendritic cell	Phase 1: Pancreatic cancer who underwent resection after initial diagnosis and then received chemotherapy	No serious side-effects were observed, except grade I fever in five and grade I reactions at the injection site in all patients	**IFN-γ ELISpot and Tetramer:** WT1-specific cytotoxic T-lymphocytes were detected in 7/8 patients.	Yanagisawa et al. (2018)
WT1-DC	WT1 peptide-loaded dendritic cells (DCs) and OK-432 adjuvant combined with molecular targeted therapy or conventional chemotherapy	Phase I/II: Metastatic or relapsed RCC/bladder cancer	2-arm: (i) WT1 peptide-pulsed DC and OK-432 adjuvant in combination with molecular targeted therapy (ii) Conventional chemotherapy No severe adverse events related to vaccination were observed; 7/10 stable disease, 3/10 disease progression	**WT1 HLA-tetramer assay:** A significant increase in the positivity of WT1-specific CD8+ T cells was observed in SD patients after DC vaccination. **IFN-γ ELISpot:** Marked augmentation of WT1-specific response by DC vaccination was observed **Flow cytometry:** WT1 specific immunity and the reduction of regulatory T cells	UMIN 000027279 (Ogasawara et al., 2018)
WT1 peptide-pulsed DC	WT1 peptide-pulsed DC	Phase 1/2: Advanced breast, ovarian, and gastric cancers	Seven patients had stable disease and three patients had progressive disease.	**Tetramer:** Tetramer-positive WT1-specific CTLs were significantly raised following DC vaccination. **IFN-γ in ELISpot:** WT1- specific CTL responses were enhanced in eight patients.	Zhang et al. (2019)
WT1/MUC1-DC	Wilms tumor gene 1 (WT1) peptide and Mucin 1 (MUC1)-pulsed dendritic cell (DC)	Phase 1/2a: Surgically resectable pancreatic ductal adenocarcinoma (PDA) patients.	No Grade 2 or higher toxicities were associated with DC vaccination. The overall survival (OS) and relapse-free survival (RFS) at 3-years from the time of surgical resection were 77.8% and 35.0%, respectively. 7/10 patients relapsed	**Delayed-type hypersensitivity:** Skin reaction was observed in nine of ten patients (90.0%) after vaccination. **Tetramer:** WT1-specific CTLs were detected after vaccination in 4/5 patients. **IHC:** The immunohistochemical analysis suggested a possible relationship between induction of WT1-specific cytotoxic T lymphocyte after DC vaccination and higher infiltration of CD3/CD4/CD8 lymphocytes in tumor tissues.	Nagai et al. (2020)
Wilms’ tumor 1 peptide-pulsed dendritic cell	Wilms’ tumor 1 peptide-loaded dendritic cells (DCs) and OK-432 adjuvant combined with conventional chemotherapy	Phase 1: metastatic or relapsed HNSCC	5/11 stable disease (SD) 6/11 progressive disease (PD)	**IFN-γ ELISpot**: A significant increase in the spot number was observed after DC vaccination in SD patients (*P* = 0.009). **Tetramer:** Trend toward remarkable increase in the percentage of HLA-A24 restricted WT1 tetramer + CD8+ T-cells in SD patients (from 0.09 ± 0.12% to 1.71 ± 1.87%) after DC vaccination compared with PD patients (from 0.03 ± 0.03% to 0.66 ± 1.12%). **CD107a mobilization assays**: WT1-specific CD107a + CD8+ T-cells increased significantly after vaccination only in SD patients (from 0.1 ± 0.19% to 3.96 ± 2.54%)	UMIN 000027279 (Ogasawara et al., 2019)
Sipuleucel-T + DNA vaccine	Sipuleucel-T and pTVG-HP with rhGM-CSF	Phase 2: Metastatic castration-resistant prostate cancer	2-arm: (i) Sipuleucel-T + pTVG-HP plasmid DNA vaccine (ii) Sipuleucel-T alone No treatment-associated events > grade 2 were observed	**ELISpot for IFN-γ and granzyme B:** Th1-biased PAP-specific T-cell responses were detected in 11/18 individuals **ELISA:** Higher titer antibody responses to PAP were detectable in patients who received pTVG-HP booster immunizations.	NCT01706 458 (Wargowski et al., 2018)
TriMixDC-MEL	DCs were harvested and co-electroporated with TriMix-mRNA (CD40L, CD70, and caTLR4 encoding mRNA) and mRNA encoding one of four TAAs MAGEA3, MAGE-C2, tyrosinase, or gp100) linked to an HLA class II targeting signal	Phase 2: Stage III/IV melanoma patients	2-arm (i) TriMixDC-MEL (ii) Standard of care Free of disease was detected in arm (i): 71%, arm (ii): 35% After a median follow-up of 53 months (range 3–67), 23 patients experienced a non-salvageable melanoma recurrence arm (i) *n* = 9 arm (ii) *n* = 14 No grade ≥ 3 AE’s occurred	**mRNA expression profiling:** STAT2, TPSAB1, CD9 and CSF2 as potential predictive biomarkers.	Jansen et al. (2020)
Audencel/Trivax	Glioblastoma dendritic cell vaccine	Phase 2: Glioblastoma multiforme	2-arm: (i) Trivax, temozolomide, surgery, radiotherapy (ii) Temozolomide, surgery, radiotherapy Failed to reach an improvement of survival	**IFN-γ ELISpot:** Post-vaccination levels of ELISPOT IFN-γ and CD8+ cells in the blood were indicative of a significantly better survival	NCT01213407 (Erhart et al., 2018)
α-Type-1 DC vaccine	Peptide-cocktail-pulsed α-type-1 DC vaccines	Phase 2: Glioblastoma multiforme (GBM)	5/16 patients were still alive, and two of these patients were relapse-free.	**IFN-γ ELISpot:** 10/15 showed positive CTL responses	Mitsuya et al. (2020)
Lipovaxin-MM	A multi-component dendritic cell-targeted liposomal vaccine	Phase 1: Metastatic melanoma	Higher grade AEs and DLTs were not observed.	**Delayed-type hypersensitivity:** Positive DTH responses were not observed **IFN-γ ELISpot:** Significant cellular and humoral immune responses were not detected in the blood of study subjects.	Gargett et al. (2018)
GM.CD40L. CCL21	The vaccine will be made by mixing two kinds of cells: (1) some lung cancer cells, which have been grown in the lab, and (2) experimental “bystander (present but not taking part in the immune response)” cells. All the cells in the vaccine will be treated with high-dose X-rays to make sure that none of them grow and cause more cancer. The bystander cells are human cells that have been genetically changed to express GM-CSF and CD40L.	Phase 1/2: Patients with Stage IV Adenocarcinoma of the Lung	Phase 1/2 (2-arm) (i) GM.DCD40L (ii) GM.CD40L.CCL21 During phase I, no dose-limiting toxicities was shown in three patients who received GM.CD40L.CCL21. Median overall survival was 9.3 versus 9.5 months with GM.DCD40L versus GM.DCD40L.CCL21 (95% CI 0.70–2.25; *p* = 0.44).	**IFN-γ ELISpot:** No significant associations between vaccine immunogenicity and outcomes,	NCT01433172 (Gray et al., 2018)
gmDC	Modified DC (gmDC) vaccine: The adenovirus (Ad-siSSF) delivers two tumor-associated antigens (TAAs), survivin and MUC1; secretory bacterial flagellin for DC maturation; and an RNA interference moiety to suppress SOCS1.	Phase 1: Relapsed acute leukemia after allogeneic hematopoietic stem cell transplantation	Two stage Phase 1: Stage 1: gmDC treatment vs. standard donor lymphocyte infusion Stage 2: gmDC vaccine only In stage 1, gmDC is safe and improved survival rate. In stage 2, observed a complete remission rate of 83% in 12 relapsed AML patients. No grade 3 or grade 4 graft-versus-host disease incidence was detected in any of the 35 patients enrolled.	**qRT-PCR:** WT1 expression was undetectable in 83% (10 of 12) of patients after Ad-siSSF-DC treatment.	NCT01956630 (Wang et al., 2018)

### Viral-Based Vaccine

As cancer vaccines targeting tumor associated antigen or protein often induce central tolerance where clinical benefit may be limited (Jou et al., 2021), packaging the antigen of interest into oncolytic viruses is known to improve its immunogenicity and efficacy. Genetically modified oncolytic viruses can replicate selectively in tumor cells and promote tumor cell lysis, at the same time promote both the innate and adaptive immune responses (Harrington et al., 2019). In 2015, FDA approved the first oncolytic virus therapy, Talimogene laherparepvec (T-VEC) for the treatment of advanced melanoma that cannot be removed by surgery. This approval was based on a Phase III OPTiM study that demonstrated significant improvement in durable response rate and overall survival when compared to granulocyte macrophage colony-stimulating factor (Andtbacka et al., 2015, 2019). In addition to viral vaccine monotherapy, a combination of viral vaccine with other approved treatments have been studied extensively in early phase clinical trials in the past few years. For example, the use of the modified vaccinia ankara (MVA) virus expressing p53 is now used in combination with chemotherapy to treat ovarian cancer and with pembrolizumab to treat advanced breast/pancreatic/liver/head and neck cancer (Hardwick et al., 2018; Chung et al., 2019). Further, MVA is engineered to expressed oncofetal antigen 5T4 (MVA-5T4, TroVax) or simultaneously expressed tumor antigen MUC1 and immune modulator IL2 (TG4010) for the treatment of metastatic colorectal cancer and advanced non-small cell lung cancer respectively (Scurr et al., 2017; Tosch et al., 2017). The use of replication-deficient human type 5 recombinant adenovirus (Ad5) vaccine to express target of interest has gained a lot of interest lately as it is the vector of choice for many COVID-19 vaccines. Ad5 is used to expressed guanylyl cyclase C (GUCY2C) fused to a CD4+ T cell epitope (the pan-DR epitope, PARDE) that is selectively expressed by intestinal epithelial cells to treat colon cancer (Snook et al., 2019). Table 4 presents an overview of the viral-based vaccine that have resulted in a publication from 2016 to 2020.

**TABLE 4:** Viral-related vaccines.

**Name**	**Composition**	**Indication**	**Trial design (single-arm unless otherwise is stated) and outcome**	**Immune monitoring method/outcome**	**References**
Ad5-GUCY2C-PADRE	Ad5-GUCY2C-PADRE is a replication-deficient human type 5 recombinant adenovirus (Ad5) vaccine encoding guanylyl cyclase C (GUCY2C) fused to the Pan DR epitope (PADRE)	Phase 1: Stage I/II colon cancer	0/10 developed adverse events greater than grade 1	**ELISA:** 10% of patients developed antibody response against GUCY2C **IFN-γ ELISpot against GUCY2C**: 40% developed GUCY2C-specific T-cell responses	NCT01972737 (Snook et al., 2019)
ACAM2000	Thymidine kinase (TK)-positive strain of vaccinia virus	Phase 1: Advanced solid tumors or acute myeloid leukemia (AML).	No serious toxicities (>grade 2) were reported	**Cytokine analysis/ELISA:** No major increase in cytokine levels	ISRCTN#10201 650) (Minev et al., 2019)
p53MVA	Modified vaccinia ankara vaccine delivering wild-type human p53 (p53MVA) in combination with gemcitabine chemotherapy in patients with platinum-resistant ovarian cancer.	Phase 1: platinum-resistant ovarian cancer.	There were no DLTs, but 3 of 11 patients came off study early due to gemcitabine-attributed adverse events (AE). Minimal AEs were attributed to p53MVA vaccination.	**Flow cytometry:** Enhanced *in vitro* recognition of p53 peptides	NCT02275039 (Hardwick et al., 2018)
p53MVA	p53-expressing modified vaccinia Ankara virus + pembrolizumab	Phase 1: Advanced breast, pancreatic, hepatocellular, or head and neck cancer	3/11 patients remained with stable disease	**Flow cytometry:** 2/3 patients showed increased frequencies and persistence of p53-reactive CD8+ T cells. Borderline or undetectable p53-specific T cell responses in 7/11 patients were related to no immediate clinical benefit.	NCT02432963 (Chung et al., 2019)
TG4010	Modified vaccinia ankara viral vaccine encoding human mucin 1 and interleukin-2 (MVA-MUC1-IL2)	Phase 2: Advanced non-small cell lung cancer.	2-arm: (i) MVA-MUC1-IL2 in combination with 1st line chemotherapy (ii) 1st line chemotherapy without MVA-MUC1-IL2 combination No grade 3–4 adverse events nor serious adverse events were considered related to TG4010. PFS was 5.1 months and 5.9 months respectively in the placebo and TG4010 arm; overall survival (OS) was 10.6 and 12.7 month in the placebo and TG4010 arm, respectively	**Tetramer:** Development of CD8+ T cell response to MVA-specific epitopes were more frequently observed in the TG4010 arm in comparison to the placebo arm.	NCT00415818 (Tosch et al., 2017)
Modified vaccinia ankara-5T4 (TroVax)	MVA-5T4, metronomic low-dose cyclophosphamide, or a combination of both treatments.	Phase 1/2: Inoperable metastatic colorectal cancer	4-arm: (i) Watch and wait (ii) Cyclophosphamide only (iii) MVA-5T4 only (iv) Combination of MVA-5T4 and cyclophosphamide No grade 3 or 4 adverse events were observed. Cyclophosphamide depleted regulatory T cells in 24 of 27 patients receiving MVA-5T4, independently prolonging PFS (5.0 vs. 2.5 months; hazard ratio [HR], 0.48; 95% CI, 0.21–1.11; *P* = 0.09).	**ELISA:** The 5T4-specific antibody immune responses were significantly increased in the MVA-5T4 (83.41 [36.09] relative units [RU]; *P* = 0.02) and combination treatment (65.81 [16.68] RU; *P* = 0.002) groups compared with no treatment (20.09 [7.20] RU).	ISRCTN54669986 (Scurr et al., 2017)
LV305	Modified, third-generation, non-replicating, integration-deficient lentivirus-based vector designed to selectively transduce dendritic cells *in vivo* expressing NY-ESO1	Phase 1: Sarcoma and other solid tumors expressing NY-ESO-1	No dose-limiting toxicities were observed. All treatment-related adverse events were grade 1 or 2. The disease control rate was 56.4% in all patients and 62.5% in sarcoma patients	**ELISpot/flow cytometry (ICS):** LV305-induced CD4+ or CD8+ T cells were detected by ELISPOT and/or ICS in 52% of patients (57% of sarcoma patients) at 1 timepoint on the study, and 18% at 2 timepoints. Induction of an anti-NY-ESO-1 immune response was associated with improved 1-year survival in an exploratory analysis.	NCT02122861 (Somaiah et al., 2019)

**FIGURE 1:**
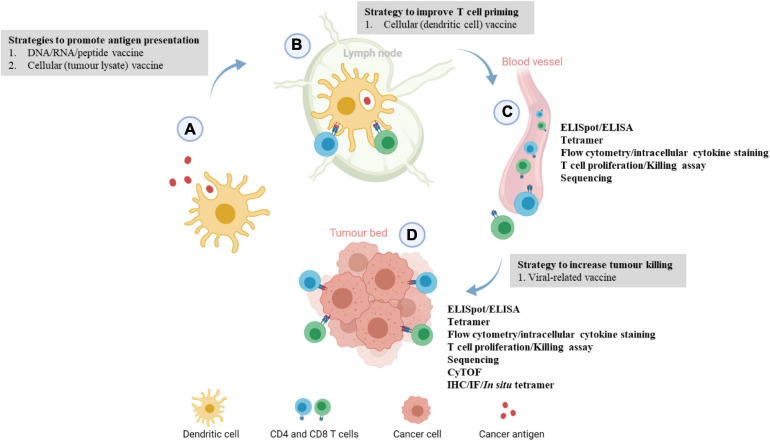
Immune monitoring of cancer vaccination at different phases of the cancer immunity cycle. **(A)** Upon vaccination, peptides or cancer antigens are digested and internalized by antigen-presenting cells, usually dendritic cells (DCs). **(B)** Antigen-presenting DCs travel to the lymph nodes to prime and activate naïve T cells through T cell receptor signaling and co-stimulation. **(C)** Upon activation, antigen-specific CD4 or CD8 T cells circulate through the blood to locate the tumor, this juncture allows the development of *ex vivo* immunoassays where antigen-specific T cells can be detected. Blood/peripheral blood mononuclear cell from cancer patients are usually harvested and antigen-specific immune responses are evaluated by ELISpot, T cell proliferation, killing assay, intracellular cytokine staining, and flow cytometry phenotyping. **(D)** At the tumor bed, activated immune cells will bind to tumor cells expressing the target of interest and induces cell lysis. Localization and levels of infiltrating antigen-specific T cells can be examined through multiplexed IHC/IF and ELISpot assay. More recently, tumor-infiltrating lymphocytes from patients’ tumors are extracted to evaluate the phenotypic changes or functions of the immune cells via RNA sequencing or scRNA sequencing. This image was created with BioRender.com.

The ultimate aim of a cancer vaccine is to generate sufficient T-cell responses. Upon antigen exposure, naïve T cells will be differentiated into effector T cells. The immune cells can be harvested while they are traveling to the tumor bed through blood vessels or when they have reached the tumor bed as indicated in Figure 1. The quantity or functionality of these antigen-specific T cells are the most important immunogenicity and efficacy readouts. Patients who ultimately demonstrating clinical benefit are those who have elevated specific immune responses. Referring to the immune monitoring methods as detailed in Tables 2–4, we will specifically discuss some of the common techniques to quantify antigen-specific T cells, and discuss their respective advantages and disadvantages in the following sections.

## Methods for Immune Monitoring

### Enzyme-Linked Immune Absorbent Spot (ELISpot)

When CD8 T cell encounters a pathogen or an antigen it recognizes, it becomes activated. This activated T cells will then secrete cytokines like TNF-α, IFN-γ, perforin or granzyme that have anti-microbial or anti-tumor effects. The enzyme-linked immune absorbent spot (ELISpot) is an assay to detect the frequency of cytokine secretion in different immune types especially T cells. In brief, T cells or co-culture of immune cells stimulated with a stimulant (antigen/peptide) will be seeded on a PVDF or nitrocellulose membrane in a 96-well plate precoated with an antibody specific to the secreted cytokine. Secreted cytokine will be captured and further detected using biotinylated antibody. This technique is regarded as a gold standard for quantitating antigen-specific cellular responses after vaccination in clinical trials (Ranieri et al., 2014), where every single spot represents a single T cell that is activated. The most widely used ELISpot in vaccine studies is the IFN-γ assay by which the secretion is augmented following immune activation, hence representing a good biomarker of successful vaccination. This assay is robust (low intra-assay, inter-assay and inter-operator variability) (Barabas et al., 2017), economical and can be conducted in a high-throughput manner. Precoated plates for different species including human, mouse and non-human primates are commercially available. Importantly, the seeded immune cells can be recovered for *in vitro* stimulation assay or other relevant assays to corroborate the findings from ELISpot reading (Saade et al., 2012). Disadvantages of ELISpot assay include the lack of information about cell phenotype and it is normally a single parameter readout. To circumvent the limitation of ELISpot assay, dual and triple color fluorospot assays allow an analysis of multiple cytokines, and commercial kits are now available (Calarota and Baldanti, 2013). Currently, several new techniques, for example, Luminex, LegendPlex and Meso-Scale Discovery (MSD) systems are being used to quantify multiple cytokines simultaneously in the cell culture supernatant, tissue homogenate, plasma, or serum. However, due to the high running cost, these are not as widely used as compared to ELISpot.

### Intracellular Cytokine Staining (ICS)

Another method to detect cytokine secretion by immune cells is intracellular cytokine staining (ICS) followed by flow cytometry analysis. This technique involves culturing immune cells in the presence of Golgi block to inhibit intracellular protein transportation. These proteins will then be retained intracellularly and therefore are available for antibody staining after fixation and permeabilization (Jung et al., 1993; Prussin and Metcalfe, 1995; Barabas et al., 2017). ICS allows both single and multiparameter cytokine analysis. In addition to detect specific cytokines, this can be coupled with markers to define specific cellular subtypes such as effector cells or memory T cells, which is not possible with the ELISpot assay (Lovelace and Maecker, 2011). This multiplexing is possible with the availability of flow cytometer that simultaneously analyzes 18 or more parameters. However, a detailed selection of antibody panel coupled with appropriate fluorochrome and careful compensation is required to ensure accurate readouts. Sometimes, signal spillover issues could compromise detection especially when there is a need for high-resolution sensitivity for example when detecting rare cell types. ICS has good sensitivity but will not be able to detect very low frequency responses as compared to the ELISpot (Karlsson et al., 2003; Maecker et al., 2005). Furthermore, ICS is more labor extensive, involves a higher cost per sample and required more expertise in detailed data analysis compared to ELISpot (Freer and Rindi, 2013). Notably, assay variability is a challenge as standardization across instruments is difficult and variability could also be introduced by different data processing methods and subjective interpretation of the researchers.

### Tetramer/Multimer Assay

In addition to quantitating the cytokine secretion by activated T cells, quantitating the expression level or the number of antigen-specific T cells using tetramer/multimer assay is also a widely used method to measure immune activation. Tetramers are synthetic structures of HLA molecules, four or more identical versions of which are linked together to form a multimeric complex that is then loaded with antigen-specific peptide (Altman et al., 1996). Antigen-loaded tetramer will be used to bind the T-cell receptors (TCR) of antigen-specific T cells, allowing the detection and quantification of this rare class of T cells through fluorescent molecules conjugated to the tetramer structure.

The major advantage of tetramer staining is the reproducibility of the assay on either fresh or cryopreserved samples when compared with ICS and ELISPOT (Maecker et al., 2008); Further, the assay time is also relatively shorter compared to ICS and ELISPOT. The downside of tetramer assay is that (i) these molecules are peptide-MHC/HLA specific, only T cells recognizing a specific MHC-peptide pair can be detected by a single tetramer. This potentially underestimates the total immune response to a pathogen or vaccine that could lead to an over interpretation of results for that particular epitope (Saade et al., 2012; Abdelaal et al., 2019); (ii) limited availability of tetramer for specific MHC molecules has limit the detection of antigen specific T cells especially in patient harboring rare MHC alleles (Abdelaal et al., 2019); (iii) tetramer is a custom-made molecule and is not available off the shelf, expertise in developing this is needed; (iv) the affinity threshold required for staining peptide-MHC complex with tetramer is higher than what is required for actual T cell activation *in vivo*, which biases tetramer to detect high affinity T cells but missing low affinity responses that could also have contributed to the immune responses (Martinez et al., 2016; Rius et al., 2018); (v) tetramer staining can inform on the phenotypes of antigen specific T cells but is unable to indicate the specific tissue location of these cells as this protocol is applied on harvested immune cells and not directly on tissue specimens (Abdelaal et al., 2019).

The ability to expand antigen-specific T cells *in vivo* is one of the direct measurements of successful cancer vaccination. However, to accurately quantitate the differences of this rare population before and after vaccination is challenging. Different innovations have been reported to increase the sensitivity of tetramer staining. Recently, there is a report demonstrating the use of magnetic nanoparticles that presented neoantigen-loaded MHC tetramers by barcoded DNA linkers which have shown to enhance the T cells-capture. The presence of magnetic nanoparticles allows the isolation of these antigen-specific T cell populations and DNA barcoding allows the multiplexing detection of different antigen-specific T cells (Peng et al., 2019).

### *In situ* Tetramer Staining

One of the limitations of the tetramer staining described in the previous section is the lack of information on the localization of the immune cells. The spatial information is gaining more importance as the presence of immune cells that can travel to the tumor bed is of therapeutic and prognostic significance (Tumeh et al., 2014). Recently, the development of *in situ* tetramer staining has provided information on the location of antigen-specific T cells and insights on the mechanisms of the anti-tumor response. This is achieved by staining tissues (FFPE or frozen section) with tetramer, followed by anti-CD8 antibody and subsequently these are detected through secondary antibodies conjugated with specific fluorophores. One of the advantages of *in situ* tetramer staining is that the signal intensity can be amplified using a relevant secondary antibody, hence lesser tetramer is needed when compared to the direct staining method, making it more cost-effective compared to direct tetramer staining (Skinner and Haase, 2002). However, *in situ* tetramer staining involves a multi-step procedure that is more time-consuming. Ideally, *in situ* tetramer staining should be performed using unfixed, fresh tissue sections to maintain the structure and mobility of T cell receptors to interact with tetramer molecule (Haanen et al., 2000; Skinner et al., 2000). Recently, this technique was used to determine antigen-specific CD8 T cells in melanoma patients post-administration of a dendritic cell vaccine and revealed the location of where these antigen-specific T cells resided (De Vries et al., 2007).

### Mass Cytometry (CyTOF)

One of the drawbacks of tetramer staining is the number of phenotypic markers that can be included in the assay. Although some flow cytometers can analyze more than 18 markers simultaneously, assay compensation has proven to be difficult. Time of flight (TOF) mass cytometry allows the evaluation of more than 40 markers per cell. Instead of labeling with fluorescently conjugated antibodies, cells are labeled with different metal isotope-tagged antibodies. When running samples on the dedicated mass cytometer, each cell is vaporized and metal ions are resolved to produce a mass spectrum. Each unique metal ion relates to the specific markers like an emission spectrum does in flow cytometry. The advantage of CyTOF stems from the minimal overlap in metal signals hence afford for detecting more than 40 parameters in a single cell resolution (Spitzer and Nolan, 2016). The disadvantages of this technique include lower flow rate as compared to flow cytometer; cells are destroyed by ionization and cannot be recovered for further analysis and the cost of running is much more compared to flow cytometry (Palit et al., 2019). Notably, the data generated by CyTOF is more complex and multidimensional and special software and expertise are needed for data analysis.

### Proliferation Assay

In addition to cytokine secretion, immune cell proliferation upon stimulation by antigen or peptide is an indication of successful cellular immunity. For example, the ability of PBMC to proliferate upon stimulation with antigen or peptide in culture has been used as a measurement of immunogenicity. In a previous section, we described the detection of immune cell expansion by quantitating antigen-specific T cells using tetramer staining. In this section, we will describe the use of dye incorporation method to track the proliferation of immune cells. Traditionally, cell proliferation is measured using the incorporation of radioisotope 3H-thymidine and this was regarded as the gold standard for measuring proliferative responses (Goodell et al., 2007). However, the use of radioisotopes requires additional safety measures and safety containment that is not available in many laboratories and 3H-thymidine incorporation assays do not provide information on the identity or function of the proliferating cells. Recently, carboxyfluorescein succinimidyl ester (CFSE) assay has been widely used to evaluate antigen-specific T-cell proliferation (Lyons, 2000). CSFE assay has eliminated the need for radioactivity and has greater flexibility, where additional antibodies can be added to identify the immune cell type that is expanding and the corresponding cytokines produced by these cells through flow cytometry analysis. PBMC can be easily stained with CFSE and T-cell proliferation following antigen stimulation is measured through the halving of fluorescence in daughter cells for 7–8 cell division. Importantly, CFSE dye incorporation can be used to track the proliferation and migration of lymphocytes *in vivo* (Parish et al., 2009). However, CFSE assays require careful optimization as a high concentration of CFSE is toxic to cells (Last’ovicka et al., 2009). Further, this assay has a high degree of variability which makes reproducibility challenging (Leroux-Roels et al., 1994). This is mainly due to differences in initial cell count, media and culture conditions (Ten Brinke et al., 2018).

### Killing Assay

In the previous sections, we have described assays to measure immune activation post-exposure to antigen/peptide, through measuring the expansion of immune cells and secretion of cytotoxic cytokines. During the antigen stimulation process and in the presence of co-stimulatory signals and CD4 helper T cells, precursor T cells will also differentiate into specialized T cells, the cytotoxic CD8 T cells (Saade et al., 2012). These cytotoxic T cells can kill target cells through two mechanisms, first is to release cytokines including perforin and granzymes, these enzymes will enter target cells and trigger a caspase cascade that eventually causes cells to undergo apoptosis; the second mechanism is through the interaction of target cell death-receptors such as Fas leading to apoptosis (Krähenbühl et al., 1988; Lowin et al., 1994). This induction of apoptosis post-exposure to antigen/peptide has made the quantification of target cell death a meaningful measurement of a successful cancer vaccine therapy *in vitro*. Traditionally, chromium (^51^Cr) release assay was used to quantify cell death, where chromium is released after the cytotoxic T cells induce lysis of ^51^Cr-labeled target cells loaded/expressed with an antigen of interest. Despite being a gold standard for measuring cell death, the use of radioisotopes is slowly being phased out due to safety reasons. Further, high interexperiment variability in labeling efficacy and high background due to the non-specific release of ^51^Cr from target cells are also some drawbacks of this technique (Zaritskaya et al., 2010).

Several non-radioactive tests have been developed to measure cell killing activity as alternatives to the chromium release assay. One of the widely used methods is the colorimetric measurement of lactate dehydrogenase (LDH) activity in the culture medium. LDH is present in the cytoplasm of all eukaryotic cells and is released into the culture media after cell death (Korzeniewski and Callewaert, 1983). LDH assay is easy to perform and has low inter-operator variability as it generates output through absorbance reading and does not involve sophisticated data interpretation. However, phenotypic identification of antigen-specific CD8 T cells and other parameters is not possible.

### RNA Sequencing

Moving into the high-dimensional analysis, RNA sequencing is a technique used to profile the cellular transcriptome quantitatively. Owing to the significant cost reduction over the years and the high throughput approach of this platform, RNA sequencing is now a viable approach in evaluating T cell responses in immunotherapy studies. Bulk RNA sequencing is widely used to characterize the cellular composition of immune infiltration (Chen et al., 2016; Sade-Feldman et al., 2019). The gene expression of immune cells within a tumor can inform us of the presence or absence of key immune cell types such as T cells, B cells, dendritic cells, natural killer cells and regulatory T cells. Additionally, bulk RNA sequencing analysis enables us to identify gene signatures that are associated with response to immunotherapies (Rooney et al., 2015; Ayers et al., 2017; Hugo et al., 2017; Cristescu et al., 2018). This knowledge will help to provide mechanistic insights into therapeutic cancer vaccines and inform on patient selection criteria for the use of cancer vaccines in the clinic. For example, RNA sequencing on PBMC samples of pediatric glioma patients revealed a response biomarker of a peptide-based vaccine where low expressions of IDO1 and PDL-1 before treatment were associated with prolonged progression free survival and patients with elevated T cell activation markers followed by cytotoxic T cell signatures are those demonstrated clinical response (Müller et al., 2018). Importantly, the vast information resulting from sequencing will provide information on possible mechanisms of treatment resistance that are not/least possibly made by all methods described previously. Challenges of RNA sequencing include the need for fresh tissue or body fluid to achieve high-quality RNA (Gallego Romero et al., 2014), and the possible generation of biases and artifacts introduced from the various sample preparation strategies (Ozsolak and Milos, 2011).

### Single Cell RNA Sequencing (scRNA-Seq)

More recently, single-cell RNA sequencing (scRNA-seq) technology has been developed to enable transcriptomic profiling analysis that is beyond the scope of bulk RNA sequencing. A major limitation of bulk RNA sequencing is that the sequence library represents the entire cell population within the tumor which obscures the heterogeneity of the transcriptomic data. Unlike bulk RNA, scRNA-seq can isolate and capture transcripts from single cells leading to the generation of sequence libraries that can be mapped to individual cells. scRNA-seq platform is currently used as a tool to examine the phenotype of key immune pathways in cancer patients. For instance, single-cell transcriptomic profiling revealed the activation and exhaustion state of infiltrated CD8 T cells in liver cancer patients (Zheng et al., 2017). Notably, the use of scRNA-seq has successfully identified a subpopulation of cells known as immunotherapy persister cells (IPCs) that resisted CD8+ T cell-mediated killing, contributing to the immune escape from anti-PD1 treatment (Sehgal et al., 2021). scRNA-seq can be applied for a more in-depth analysis of the immune cell’s functionality as opposed to only focusing on immune cell composition achieved through the conventional bulk RNA sequencing. Additionally, the use of scRNA sequencing platform will also provide the opportunity to characterize rare or unknown immune cell types or signatures that may be associated with therapeutic responses. Currently, published data on single cell transcriptomic responses in the context of therapeutic cancer vaccination are still lacking and there remains much to learn from other available pilot studies on data analysis and interpretation.

### TCR Sequencing

The T cell receptors (TCRs) on the surface of T cells are responsible for recognizing antigens presented by the major histocompatibility complex in human. The availability of RNA sequencing technologies has made it possible to profile T cell receptors (TCR) at the transcriptomic level. The TCR repertoire is one of the predictive biomarkers that is associated with effective immunotherapy responses. For example, the TCR repertoire of immunotherapy-treated pancreatic ductal adenocarcinoma was shown to be associated with a patient’s survival (Hopkins et al., 2018). In response to antigen stimulation, T cells will undergo proliferation and clonal expansion. Therefore, identifying vaccine-responsive T cell clonotypes is informative for the monitoring of therapeutic cancer vaccines. In a trial evaluating the combination of dsRNA analog and tumor-lysate loaded dendritic cells vaccine, TCR sequencing performed on the patient’s PBMC indicated some clones to be enriched 42 days post-vaccination, suggesting the clonality of T cells was induced by the immunotherapy (Rodríguez-Ruiz et al., 2018). A study also demonstrated patients who underwent tremelimumab treatment have broadened their TCR diversity, suggesting the use of CTLA4 checkpoint blockade has expanded the pool of circulating T cells (Robert et al., 2014). Additionally, TCR sequencing is also used to evaluate the infiltration of functional peptide-specific cytotoxic T cells through the detection of an identical sequence of peptide-specific TCRs from T cells derived from both tumor tissue and PBMC (Mackensen et al., 1997; Daiko et al., 2020). Importantly, TCR sequencing has also revealed that patients who responded to checkpoint blockade are those with less clonotype loss during treatment (Cha et al., 2014). TCR sequencing is the only available technique that can provide insights into the T cell clonotypes and diversity post immunotherapy treatment. The disadvantages of this technique are mainly due to the inconsistency of input materials (DNA, mRNA or PCR enriched DNA) and variations resulting from different data analysis pipelines (Liu and Wu, 2018).

## Summary

In summary, when a naïve T cell encounters its cognate antigen, it will get activated and differentiate into specialized T cells, followed by clonal expansion. These specialized T cells will then migrate from the lymph node to tumor bed, where they will hunt and kill target cells expressing the same antigen. The induction of this series of processes represents an active stimulation by a cancer vaccine. The ability to detect these processes individually has provided insight into successful vaccinations. In this paper, we have discussed 10 different techniques that are currently being used to evaluate immune responses triggered by cancer vaccination in both preclinical and clinical studies. As clinical immune monitoring is a continuous process, despite the advances in new technologies, ELISpot and flow cytometry are still the preferred methods due to their affordability and robustness. Other methods such as *in situ* tetramer staining and sequencing (RNA, sc-RNA, and TCR sequencing) are currently used in the discovery setting rather than as a clinical monitoring tool.

Important factors that need to be taken into account for the successful detection and measurement of T-cell immunity in response to immunization are the timings of when each assay is performed and when samplings are made. Worth noting that immune response is a dynamic response, different vaccine candidates will confer different kinetic profiles. It is advisable to conduct a timepoint measurement to identify a time point that confers the highest immune activity before a study is implemented. In some instances, even at the time of maximum response, the frequency of antigen-specific T cells in the peripheral blood may be below the limits of detection (Saade et al., 2012). Despite the advancement in detecting immune responses at single-cell level, defining T cell responses that are reflective of conferring protection remains the biggest challenge for the field.

## Author Contributions

KPL contributed to the concept and manuscript writing. NSZ contributed to the manuscript writing. Both authors contributed to the article and approved the submitted version.

## Conflict of Interest

The authors declare that the research was conducted in the absence of any commercial or financial relationships that could be construed as a potential conflict of interest.
